# Cardiac surgery and percutaneous intervention in pregnant women with heart disease

**DOI:** 10.1007/s12471-012-0244-3

**Published:** 2012-02-21

**Authors:** P. G. Pieper, E. S. Hoendermis, Y. N. Drijver

**Affiliations:** Department of Cardiology, University Medical Centre Groningen, University of Groningen, PO Box 30.001, 9700 RB Groningen, the Netherlands

**Keywords:** Pregnancy, Heart disease, Cardiac surgery, Cardiopulmonary bypass, Catheter intervention

## Abstract

In pregnant women with heart disease, complications can arise due to the haemodynamic burden of pregnancy and to hypercoagulation. Most problems can be managed medically, but sometimes cardiac surgery or percutaneous intervention is unavoidable. Cardiac surgery has similar maternal mortality to that outside pregnancy, but foetal mortality and morbidity are considerable. Measures to reduce the risk by adaptation of the management of cardiopulmonary bypass are described. When gestational age is > 28 weeks, pre-surgery delivery of the foetus should be considered. Percutaneous intervention exposes the foetus to radiation. The radiation dose for common cardiac procedures, however, does not result in detectable harmful foetal effects.

## Introduction

Heart disease is an increasingly important cause of maternal morbidity and mortality during pregnancy. Most cardiac problems that arise in pregnant women with heart disease can be managed without interventional procedures. However, the haemodynamic changes of pregnancy (increase in circulating volume and cardiac output, decrease in vascular resistance, and hypercoagulation) may sometimes lead to deterioration in previously stable women. Indications for intervention may arise when cardiac conditions worsen during pregnancy (such as rapid progression of aortic dilatation in Marfan syndrome), when the severity of the disease was undiagnosed or underestimated before pregnancy (which is often the case in mitral stenosis), or when new complications or diagnoses arise (for example prosthetic valve thrombosis, endocarditis or myocardial infarction)[1].

This special article reviews the indications, timing, maternal and foetal risk and management of cardiac surgery as well as of catheter interventions in pregnant women with heart disease.

## Indications for intervention during pregnancy

Maternal mortality associated with cardiac surgery is relatively high (6%). This is probably caused by the urgent character of the procedure and mortality is not different from that in non-pregnant women undergoing similar emergency procedures [1–3]. However, because the risk of foetal loss and late morbidity for the child is considerable, cardiac surgery should only be performed when medical therapy or interventional procedures fail and the mother’s life is threatened. Percutaneous intervention exposes the foetus to the risk of radiation. The risk is limited for most procedures, but percutaneous intervention should only be performed when there are no medical therapeutic options. Since the risk for the foetus is lower than the risk of cardiac surgery, percutaneous intervention is preferred when possible [2].

### Valvular heart disease

Mitral stenosis is a frequent diagnosis in immigrants and is still prevalent in developing countries. Mitral stenosis is not well tolerated during pregnancy and pre-pregnancy intervention is recommended when the valve area is <1.5 cm^2^. When pulmonary oedema or pulmonary hypertension develop during pregnancy, therapy with β-blockers and diuretics is indicated. When, despite optimal medical therapy, the woman remains in NYHA class III/IV or systolic pulmonary artery pressure remains > 50 mmHg, percutaneous intervention can be considered. Since a short radiation time is important the procedure should be performed by an experienced operator. Abdominal shielding is advised [1, 2].

Aortic stenosis in young women of fertile age is usually caused by congenital valve malformation. Women with aortic stenosis are at increased risk of cardiac and obstetric complications [4–6]. Heart failure, arrhythmias and hypertensive disorders are the most frequent complications. The complication rate is associated with stenosis severity [4, 6]. Previously, pre-pregnancy intervention was advocated in all women with severe aortic stenosis [7]. However, although clinical deterioration during pregnancy in women with severe aortic stenosis has been described, the risk of mortality is very low according to recent series [2, 8, 9]. When women with severe aortic stenosis contemplate pregnancy, the risk of pregnancy should be weighed against the risk of pre-pregnancy surgical treatment. Biological valves have a high deterioration rate and will inevitably result in eventual re-operation, while mechanical valves are associated with a high risk of severe maternal complications [2, 7, 10]. Therefore, according to the most recent European Society of Cardiology guidelines, pre-pregnancy intervention is only advised in women with severe aortic stenosis and symptoms, an abnormal exercise test or left ventricular dysfunction, [2]. When a woman with severe aortic stenosis deteriorates during pregnancy and medical therapy fails, balloon valvuloplasty by an experienced operator may be a suitable option in non-calcified valves. Even in calcified valves, balloon valvuloplasty, although not without risk, may be a safer option than surgery, and it may serve as a bridge to surgery after delivery. However, in some cases surgery may be unavoidable[1, 2].

Regurgitant lesions, although not innocent, are better tolerated than stenotic lesions and rarely require intervention during pregnancy [4, 8].

Mechanical prosthetic valves may require surgery during pregnancy since the risk of valve thrombosis is considerable [2, 4, 10, 11].

### Aortic disease

Aortic pathology was the second cardiovascular cause of maternal mortality in the UK in the most recent report on maternal mortality [12]. Progressive aortic dilatation and aortic dissection are mainly observed in women with Marfan syndrome, Ehlers-Danlos syndrome, Turner syndrome and bicuspid aortic valve. The European Society of Cardiology guidelines recommend pre-pregnancy surgery in women with Marfan syndrome or bicuspid aortic valve when the aortic diameter is > 45 mm and > 50 mm, respectively. During pregnancy, prophylactic surgery should be considered when the aortic diameter is > 50 mm and increasing rapidly. When aortic dissection occurs during pregnancy, surgery should be performed. When the foetus is viable, caesarean section is recommended immediately before cardiac surgery [2].

### Ischaemic heart disease

Ischaemic heart disease is increasingly encountered during pregnancy due to delay of pregnancy to older age and unhealthy lifestyle. It is an important cause of maternal mortality [12]. In a recent review comprising 103 pregnant women with acute myocardial infarction, maternal mortality was 11%. Atherosclerosis was the underlying cause in 40% of infarctions. Coronary dissection occurs more often than outside pregnancy and was responsible for 27% of the cases in this series [13]. Percutaneous coronary intervention has now been reported in more than 200 cases. In women with ST-elevation myocardial infarction, it is the reperfusion therapy of choice. Experience with thrombolysis is limited during pregnancy, but it carries the risk of subplacental bleeding. Thrombolysis is also contraindicated because there is a high risk of coronary artery dissection as the underlying mechanism of the infarction [1, 2]. Percutaneous intervention has reduced the necessity for coronary artery bypass grafting during pregnancy, which is now extremely rare [1]. The risks for the foetus resulting from radiation exposure are limited but should be taken into account. Bare metal stents are preferred during pregnancy, because the safety of drug-eluting stents is unknown. The need for prolonged dual antiplatelet therapy after placement of drug-eluting stents is a concern [2].

## Cardiopulmonary bypass during pregnancy

Cardiopulmonary bypass during pregnancy is associated with similar maternal mortality to that outside pregnancy [2, 3]. However, the foetal mortality rate is 14 to 33% [1, 3, 14]. Sustained uterine contractions resulting in uteroplacental hypoperfusion and foetal hypoxia are considered the most important cause of foetal death [14]. Causative factors for uterine contractions are haemodilution, causing dilution of progesterone, as well as cooling and rewarming. Nonpulsatile flow triggers vasoconstriction, further increasing placental dysfunction [14]. Uteroplacental flow is further compromised by decrease in maternal blood pressure. Foetal bradycardia is often observed. Although the mechanism of this bradycardia is not known, foetal hypoxia due to haemodilution and to uteroplacental hypoperfusion probably plays a role [14]. In addition to foetal mortality, significant morbidity including late neurological impairment has been described, mainly associated with premature birth [2, 3]. Because of these risks, cardiac surgery should be avoided whenever possible. In the first trimester the risk of foetal malformations is higher, therefore postponement of cardiac surgery until after the 13th week of gestation is preferred. It is recommended to monitor uterine contractions and foetal heart rate during surgery. To diminish the risk of foetal bradycardia and uterine contractions, the European Society of Cardiology guidelines recommend to minimise cardiopulmonary bypass time and to maintain a pump flow > 2.5 l/min/m^2^, a perfusion pressure > 70 mm Hg, a maternal haematocrit > 28%, and to use pulsatile flow and normothermic perfusion [2]. Additionally a left lateral tilt of 15º is recommendable to relieve pressure of the uterus on the caval vein.

Because of the high foetal risk, when gestational age is advanced, it should be considered to deliver the baby before cardiac surgery. However, it is a matter of debate at which gestational age pre-surgery delivery is advantageous for the foetus, since premature birth is in itself associated with foetal mortality and morbidity. Although the prognosis of very premature babies has improved in recent years, before 26 weeks of gestation neonatal mortality is around 40% and delivery before surgery is not recommended. After 28 weeks of gestation, neonatal mortality is < 10% and severe morbidity is limited; therefore, the guidelines recommend that delivery before cardiopulmonary bypass should be considered. If possible the surgery should be delayed until a full course of corticosteroids (at least 24 h) has been administered to the mother, since this improves foetal outcome considerably [2]. Between 26 and 28 weeks the prognosis of the baby depends on gender, estimated birth weight, the presence of foetal malformations, and the administration of corticosteroids. Additionally the experience of the local neonatal unit is important in the decision whether or not to perform a caesarean section before cardiopulmonary bypass. The decision should be made on an individual basis [2]. It should be kept in mind that maternal prognosis may be negatively influenced by surgery shortly after delivery, therefore delivery should only be performed when the advantages for the baby are clear [15].

## Percutaneous interventions during pregnancy

When pregnant women are exposed to diagnostic or therapeutic procedures involving radiation, this may cause high levels of anxiety, both among the health care providers and the women. This sometimes results in termination of pregnancy. Indeed, ionising radiation can have harmful effects, which are cell death and teratogenic effects, carcinogenesis and genetic effects (mutations)[16]. These effects are, however, not observed with the doses that are needed for the majority of diagnostic and therapeutic procedures. There is consensus that the risks for the foetus are very limited when the dose to the mother is < 50 mGy [2, 16]. With doses below this level, there is no observed increased risk of foetal malformations. A very small increase in the risk of childhood cancer cannot be ruled out. The most frequently performed cardiac percutaneous interventions during pregnancy are mitral balloon valvuloplasty and coronary angioplasty. Radiation doses to the mother for such procedures are < 20 mGy. The foetal risk is limited further because the foetus is outside the field of direct radiation during cardiac procedures. Even though the risks are small, radiation exposure should be avoided when possible. However, if a procedure is absolutely necessary and there are no alternatives, it should not be withheld and the mother can be reassured. Radiation doses to the foetus should be kept as low as possible. This can be achieved by minimising radiation time. Therefore procedures should be performed by experienced cardiologists. Abdominal shielding is recommended, although this lowers the dose to the foetus by only 2%. Direct radiation of the abdominal region should be avoided as much as possible. The best period to perform invasive procedures is the beginning of the second trimester, when organogenesis is complete but the uterus is still small [2].

Iodine-containing contrast agents have been studied in animals and did not appear to be teratogenic. There is, however, a concern for neonatal hypothyroidism, therefore their use should be minimised [16].

## Summary and conclusions

Cardiac surgery is sometimes unavoidable during pregnancy. Mortality risk for the mother is 6% and is comparable to that outside pregnancy. Foetal mortality risk is however 14–33% and there is considerable foetal morbidity. Therefore, when the foetus is viable, caesarean delivery before cardiac surgery should be considered. When this is not possible, cardiopulmonary bypass should be adapted to meet the needs for adequate uteroplacental perfusion and to minimise the inducing of uterine contractions. Percutaneous intervention, mostly mitral balloon valvuloplasty and coronary angioplasty, exposes the foetus to radiation. The procedures should only be carried out when absolutely necessary, but the radiation dose associated with these interventions is not likely to cause harm to the foetus.
